# BERT-PAGG: a Chinese relationship extraction model fusing PAGG and entity location information

**DOI:** 10.7717/peerj-cs.1470

**Published:** 2023-07-17

**Authors:** Bin Xu, Shuai Li, Zhaowu Zhang, Tongxin Liao

**Affiliations:** School of Computer Science and Engineering, Northeastern University, Shenyang, Liaoning, China

**Keywords:** Relation extraction, Entity type, Segmented convolutional neural networks, Gating mechanism, Self-attention mechanism

## Abstract

Relationship extraction is one of the important tasks of constructing knowledge graph. In recent years, many scholars have introduced external information other than entities into relationship extraction models, which perform better than traditional relationship extraction methods. However, they ignore the importance of the relative position between entities. Considering the relative position between entity pairs and the influence of sentence level information on the performance of relationship extraction model, this article proposes a BERT-PAGG relationship extraction model. The model introduces the location information of entities, and combines the local features extracted by PAGG module with the entity vector representation output by BERT. Specifically, BERT-PAGG integrates entity location information into local features through segmented convolution neural network, uses attention mechanism to capture more effective semantic features, and finally regulates the transmission of information flow through gating mechanism. Experimental results on two open Chinese relation extraction datasets show that the proposed method achieves the best results compared with other models. At the same time, ablation experiments show that PAGG module can effectively use external information, and the introduction of this module makes the Macro-F1 value of the model increase by at least 2.82%.

## Introduction

Relationship extraction aims to find the semantic relationship between two related entities, which may be unrelated or related in some way. In this article, we focus on Chinese relationship extraction. For Chinese relational extraction, the linguistic and semantic environment is more complex and it typically faces two challenges: (1) wrong participle. The example sentence “化妆和服装” has the correct split result of “化妆/和/服装”, but if it is split into “化妆/和服/装”, the semantic meaning is altered, which will affect the subsequent parsing work. (2) Chinese has a more flexible grammatical structure and a more variable sentence structure. As a result, more grammatical structures and more meaningful features must be considered in Chinese relational extraction.

The traditional approach to the relation extraction task is based on deep learning to obtain information about entities in sentences *via* neural networks and subsequently perform relation extraction, but this approach does not take into account other information contained in sentences, such as sentence information, entity location information, and syntactic structure information. Another key factor in improving the relationship extraction effect is the use of this external information. For example, the Chinese sentences “奥巴马出生在美国” and “奥巴马在美国曾”, both sentences have the entities “担任总统职位” and “奥巴马”, but the relationship between the entities in the two sentences is completely different, and the relationship between the different sentences containing the same entities may be different or the same. If we simply use entity information for relationship extraction and use the same word vector for entities, it will cause difficulties in relationship classification and affect the effectiveness of relationship extraction. Therefore, how to represent entities and introduce sentence information in the relationship extraction model are the key factors to improve the performance, and good entity representation and rich sentence information can avoid the above problems.

Furthermore, it can be found from the above two sentences that the relationship between entities in Chinese sentences is mostly the content between entities, and the positional relationship between characters other than entities and entities is also a key factor affecting the performance of relationship extraction. For example, the word “美国” in the above sentence is located between two entities, and it is essential to introduce such location information. Note that the syntactic structure should also be taken into account in the relationship extraction task, since the features extracted using word sequences alone are not sufficient and cannot accurately model the relationships between entities, and a model using syntactic dependencies can capture more useful information.

In this article, we propose the BERT-PAGG (Bert-based Piecewise Convolutional Neural Networks, Self-Attention Gate and GCN model) relationship extraction model, which improves the relationship extraction by using a combination of external and entity information in order to extraction performance by using a combination of external and entity information. The main work is as follows.

1. We add “#” and “$” signs on both sides of the head and tail entities of the input text to make the BERT pre-trained model better capture inter-entity information.
2. To better capture the structural information features of the two entities, we introduce the relative positions of the entities and extract the local features of the sentences by a segmented convolutional neural network PCNN.
3. The self-attention mechanism is employed to capture global dependent features and a gating mechanism is utilized to adaptively fuse global and local features.
4. A graph convolutional neural network is employed to fuse semantic and structural information with entity-specific masks to mask the non-entity words learned by the GCN layer, followed by the use of a retrieval-based attention mechanism to extract important features of contextual words, which are finally combined with the features output by the gating mechanism for the relationship extraction task.

## Related Work

As an essential subtask in information extraction, relation extraction is also a key step in building knowledge graphs. With the emergence of deep learning, existing relationship extraction models mainly rely on neural networks to extract the semantic information of sentences.

Socher et al. (2012) combined a matrix vector representation with a recurrent neural network. The model learns a combined vector representation of phrases and sentences of arbitrary syntactic type and length. The problem that the word vector space model cannot capture long sentence information is solved. However, since RNN cannot process the long-term dependency problem and the existence of gradient explosion problem, Le et al. (2018) innovatively proposed a multichannel long short-term memory (LSTM) model, which combines multiple feature embeddings and uses BiLSTM to encode different feature embeddings to improve the adaptability and robustness.

CNN can extract local features more efficiently in shorter sentences and has a simpler structure and faster training than RNN. Zeng et al. (2014) used convolutional neural networks for the first time to extract hierarchical features of words and sentences for relationship extraction in a relationship classification task and used positional features to improve feature extraction, resulting in a significant improvement in model performance. Nguyen & Grishman (2015) used convolutional neural networks to replace the traditional feature engineering approach, using convolutional kernels of different sizes to extract local features of entity contexts and using positional embeddings to encode relative distances, resulting in a further improvement in the performance of the proposed model over the single kernel CNN. To reduce the impact of error accumulation kernel propagation, Zeng et al. (2015) improved the pooling layer of traditional convolutional neural network (CNN) to obtain PCNN (Piecewise CNN), which pools the feature map into three segments by two entity locations, with the aim of better capturing the structured information between two entities and using attention mechanism by building sentence level selective attention neural model to alleviate the mislabeling problem, these efforts improve the overall performance of the model.

As noisy signals will cause errors, in order to reduce their impact on model performance, Lin et al. (2016) applied the attention mechanism to PCNN. They used a convolutional neural network to embed the semantics of sentences and constructed sentence-level attention on instances to reduce the impact of noise by adjusting the weights. Although the PCNN with the introduction of the attention mechanism has achieved some success in the relational extraction task, it does not have good access to contextual information; therefore, Lee, gyu Seo & Choi (2019) proposed a new end-to-end recurrent neural model that combines LSTM and entity-aware attention mechanism, which enables the model to utilize contextual information and solve the long-term dependency problem with better results. While Zhou & Chen (2022) improved entity representation to reduce noise in labels and better reveal the contextual semantic representation of entities.

In order to make the model utilize more information, many scholars started to incorporate features of different granularity into the model, such as word-level features and character-level features. Zhang & Yang (2018) proposed a lattice-LSTM model, which encodes a series of input characters as well as all potential words from the matching dictionary, where the gated recurrent cell both makes full use of the word sequence information and enables the model to select the most relevant words and characters from the sentence. Xu, Yuan & Zhong (2020) merged word and character inputs and proposed a Lattice-GRU model with Bi-GRU instead of Bi-LSTM. Li et al. (2019) proposed a model for Chinese relation extraction that incorporates word-level information into character sequences and introduces external knowledge. Huang et al. (2021) used the intermediate layer of BERT to obtain different levels of semantic information and design multi-granularity features for the final relation classification. Kong et al. (2021) proposed an adaptive approach to improve the performance of Chinese relationship extraction by combining word information into the embedding layer of the character input-based model using a dictionary to address the information loss problem of the MG-Lattice model. Xue et al. (2019) proposed a focused attention model based on the BERT language model and dynamic range attention mechanism for joint entity and relation extraction tasks. The model combines the BERT language model with joint learning to improve the feature representation capability of the shared parameter layer through a dynamic range attention mechanism.

In recent years, some researchers have considered the semantic connection between entity positions. Zeng et al. (2014) introduced positional embedding, which takes the relative distance between each word and the target entity as an important input. Considering the importance of syntactic dependencies, researchers have improved LSTM, and Tai, Socher & Manning (2015) proposed TreeLSTM, through which the tree structure model is able to use the syntactic structure tree of a sentence as an input to obtain sentence features for relation extraction. The graph neural network proposed by Scarselli et al. (2009) has received wide attention from researchers, and Zhang, Qi & Manning (2018) were the first to use graph convolutional networks for the relational extraction task, constructing graphs through the dependency tree structure of sentences and performing path-centered pruning on the dependency trees, and proposing the C-GCN model based on the PA-LSTM (Zhang et al., 2017) model for the relational extraction task significant progress was achieved. Subsequently, based on the study of C-GCN by Guo, Zhang & Lu (2019) and others, the AGGCN model was proposed, where instead of pruning the dependency tree afterward as the input to the model, the model was dynamically adjusted by the Attention mechanism, by which the model was able to capture richer local and non-local information.

Since Chinese texts from different domains involve widely different relationship and entity features, and the high complexity of the Chinese language, including the complexity of language structure, semantic complexity, and vocabulary complexity, all these bring challenges to Chinese relationship extraction. Furthermore, since texts oriented to the social media domain often lack valid information, finding content beyond the text to supplement textual information is imperative, and according to the review by Zhu et al. (2023) and Zhu et al. (2022). multimodal information extraction is an important research area that many scholars have now applied to specific domains.

## BERT-PAGG model

**Figure 1:**
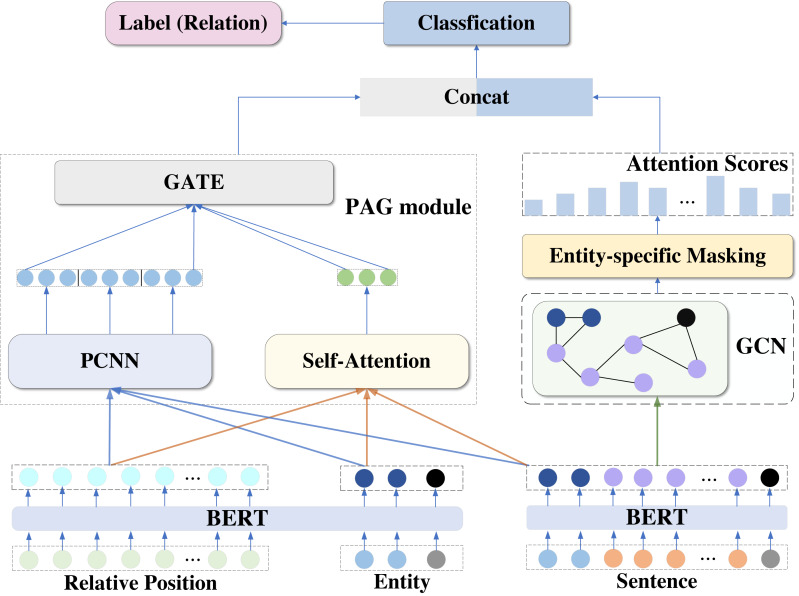
Overview of BERT-PAGG structure.

To perform better relationship extraction, the model proposed in this article introduces a segmental convolutional neural network, self-attention mechanism, gating mechanism, and graph neural network based on BERT pre-training model, and implements a BERT-PAGG relationship extraction model based on BERT. The overall structure of the model is shown in Fig. 1.

### Input layer

The input of the BERT-PAGG model mainly consists of sentences $S= \left\{ {c}_{1},{c}_{2},\ldots ,{c}_{n} \right\} $ and entity positions. Firstly, the input sequence $ \left\{ {c}_{1},{c}_{2},\ldots ,{c}_{n} \right\} $ is encoded by the BERT pre-training model to obtain $ \left\{ {e}^{{c}_{1}},{e}^{{c}_{2}},\ldots ,{e}^{cn} \right\} $ , where each word *c*_*i*_ in the sentence corresponds to the encoded *e*^*c*_*i*_^ .

### Vector representation layer

The model proposed in this article is also built on the BERT pre-training model, which can obtain richer information from the text and provide better text representation. In the relational extraction task, how to obtain entity information efficiently is the key to improving the performance of the relational extraction model. Wu & He (2019) proposed the R-BERT relational extraction model for the relational classification task, and proved that the tagging process for entities in the relational extraction model is effective. Special tags, such as “#” and “$”, are added to the head and tail entities in the input text, and such tags enable the BERT pre-training model to capture entity information better.

For the text in the dataset, special markers are added to the head and tail entities according to their positions, respectively, in the form shown in Table 1.

**Table 1:** Example of adding special tags into text.

Dataset	Sentence	Relation
FinRE	# 东方航空 #AH 股临时停牌传将与 $ 上航 $ 合并。 Eastern Airlines # Stock suspension $ Shanghai Airlines $ merge.	合并 merge
SanWen	# 旅行车 # 继续在 $ 崇山峻岭 $ 中穿行。 #station wagon # continue $ mountains$ travel through.	Located

Wu & He (2019) conducted ablation experiments for the method of adding markers to the head and tail entities, and the experimental results showed that the effect of adding markers to the head and tail entities separately is better than that of adding markers to the head or tail entities, and also better than the effect of not adding markers. Therefore, in this article, we add “#” and “$” marks on both sides of the head and tail entities of the input text, which enables the Attention mechanism of the Transformer structure in the BERT pre-training model to better capture the information of the two entities and the relationship information between them, including the relationship information between the two entities, the information between the two entities and other words, and the semantic information between the two entities.

We input the above text sequence with “#” and “$” marks and entity positions into BERT to obtain the corresponding entity vector representation, where the vector representation of the first word of the head entity and the vector representation of the first word of the tail entity are spliced to represent the vector representation of the head and tail entities as shown in Eq. (1).

(1) \begin{eqnarray*}Entity= \left[ {h}_{head};{h}_{end} \right] .\end{eqnarray*}

### Segmented convolutional neural network

**Figure 2:**
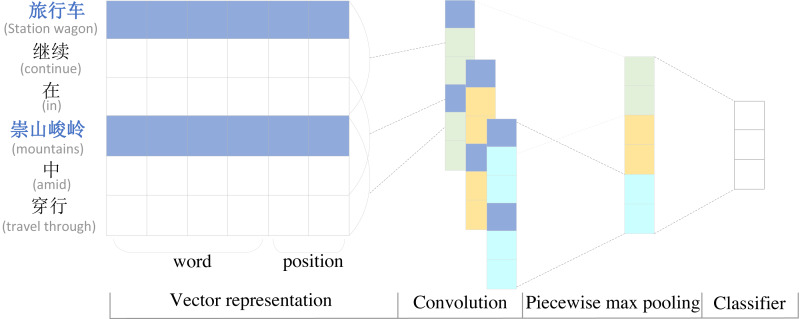
Diagram of piecewise convolutional neural network.

To highlight the importance of the first and last entities and their location information, we use a segmental convolutional neural network (PCNN) to extract sentence local features. Segmented convolutional neural network PCNN is a widely used model for relation extraction. Considering that the relative positions of words and entities also have an impact on the effect of relation extraction in the relation extraction task, the position information is also added to the encoding layer. Since the traditional single max-pooling can only get one value per convolutional kernel, which cannot capture the structural information features between two entities, PCNN introduces a segmented max-pooling mechanism, which cuts the text into three parts according to the head entity and the tail entity, extracts the local features separately and then splices the three vectors obtained by segmented max-pooling to obtain the final feature vector, This mechanism can extract the sentence feature information more effectively. The structure of the PCNN network is shown in Fig. 2.

The PCNN relationship extraction model differs from the traditional relationship extraction model by using only the word vector representation as the input of the model. Considering the importance of the positional relationship between words and entities, PCNN distinguishes entities from other words and also splices the positional features of words and head and tail entities with word embedding in the vector representation layer as the input of the final model, enabling the model to obtain rich positional information. The calculation is shown in Eqs. (2)–(4).

(2) \begin{eqnarray*}B=BERT(S)\end{eqnarray*}

(3) \begin{eqnarray*}Position=Embedding(Index)\end{eqnarray*}

(4) \begin{eqnarray*}P=PCNN(B,Position)\end{eqnarray*}

where *B* is the embedding representation of the sentence *S* after BERT encoding, *index* is the position of the word relative to the head and tail entities, the corresponding position embedding representation is obtained by *Embedding*, and finally the word embedding *B* and position embedding *Position* are stitched together and input to PCNN to get the output *P*.

Through the studies and analysis of the distance between the word and the entity in the sentence, it was found that the smaller the distance between the word and the entity, the greater the influence on the final relationship extraction results, so the relative distance between the word and the head and tail entity was used to represent the location feature in PCNN. As shown in Table 2, the head entity in the sentence is “旅行车” and the tail entity is “崇山峻岭”, and the location features of other characters in the sentence are expressed as the relative distance from the current word to the head and tail entities, which are encoded by the location representation matrix $D\in {R}^{{d}^{p}\times \left\vert {v}^{p} \right\vert }$ , where *d*^*p*^ is is the dimension of the position vector, and *v*^*p*^ denotes a fixed-size position table.

The word vector representation and the position vector representation are concatenated to form the final vector representation as the input of the model, for the input text $S= \left\{ {c}_{1},{c}_{2},\ldots ,{c}_{n} \right\} $ , the word vector representation $ \left\{ {e}^{{c}_{1}},{e}^{{c}_{2}},\ldots ,{e}^{cn} \right\} $ is obtained by BERT, and the position vector representations and are obtained by position features. The final stitching yields *SP* = [*S*; *HEAD*; *TAIL*] , where $SP\in {R}^{d} \left( d\in {d}^{w}+2\times {d}^{p} \right) $ is taken as the input to the model.

(5) \begin{eqnarray*}PCN{N}_{\text{outputs}}=\tanh \nolimits \left( {P}_{1:n} \right) .\end{eqnarray*}

**Table 2:** Relative head and tail entity position.

	旅行车	继	续	在	崇山峻岭	中	穿	行
Sample	Station wagon	Continue		In	Mountains	Amid	Travel	Through
Relative head entity position	0	1	2	3	4	5	6	7
Relative tail entity position	−4	−3	−2	−1	0	1	2	3

### Self-attention mechanism

The PCNN has the advantages of capturing local features, small computation and simple structure, and is the mainstream model in relation extraction. The contextual representation of sentences can be obtained by using PCNN, but long-term dependency features of sentences cannot be obtained by using PCNN alone, even by stacking multiple convolutional neural networks to achieve better performance. In this article, the self-attention mechanism is employed to capture global dependency features that complement the contextual representation obtained by PCNN.

Instead of using the PCNN in tandem with the self-attention mechanism in the previous way, a parallel approach is adopted in this article. For the sentence input, the contextual representation of the sentence is obtained by PCNN, the global dependent features are captured by the self-attention mechanism, and finally the global dependent features and the contextual representation are put into the gating mechanism to adaptively fuse the two features and get the final output. the PCNN has been introduced in the previous section, and the output calculation formula of the self-attention mechanism module is given here as Eqs. (6)–(8) shows.

(6) \begin{eqnarray*}Att={W}^{2}\sigma \left( {W}^{1}SP+{b}^{1} \right) +{b}^{2}\end{eqnarray*}

(7) \begin{eqnarray*}{P}^{(Att)}={softmax}\nolimits (Att)\end{eqnarray*}

(8) \begin{eqnarray*}A=\sum {P}^{(Att)}\odot SP\end{eqnarray*}

where *W*^1^ and *W*^2^ are the weight matrices, *b*^1^ and *b*2 are the biases, and the final output *A* is obtained.

### Gating mechanism

To introduce both local and global dependent features of a sentence into the relational extraction task, we need to fuse the two features. Traditional features fusion approaches (*e.g.*, feature stitching (Bell et al., 2015), feature summation (Kong et al., 2016), *etc.*) do not consider whether the participating features are useful for the final task, so the fused features may contain a lot of unwanted information. Considering that the gating mechanism based on Gated Tanh Units (GTU) and Gated Linear Units (GLU) will measure the usefulness of each feature vector in the feature map during fusion and select the information that is useful for the final task for aggregation, therefore, this article uses the gating mechanism to adaptively fuse the entity features extracted by the PCNN network and the global dependent features extracted by the attention mechanism are fed to the classification layer as a representation of the whole text for classification, and the gating mechanism is calculated as shown in Eqs. (9)–10.

(9) \begin{eqnarray*}g={sigmod}\nolimits \left( {W}^{g1}\tanh \nolimits \left( {W}^{g2}A+{b}^{g2} \right) +{b}^{g1} \right. \end{eqnarray*}

(10) \begin{eqnarray*}outputs=g\cdot PCN{N}_{outputs}\end{eqnarray*}

where *W*^*g*1^ and *W*^*g*2^ are the weight matrices, *b*^*g*1^ and *b*^*g*2^ are the biases, and finally the *outputs* is obtained through the gating mechanism.

### Entity-specific graph convolutional neural networks

#### Constructing the graph

**Figure 3:**
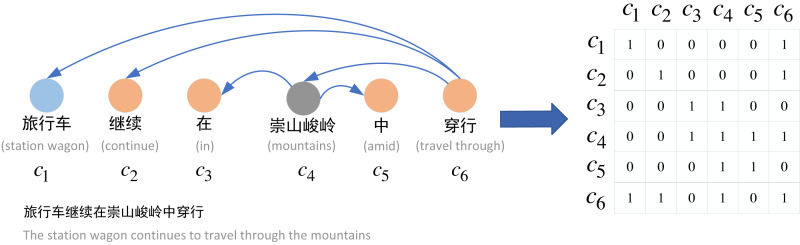
Construction of adjacency matrix.

To construct the graph neural network structure in the relational extraction task, we construct a syntactic dependency graph for each sentence. Figure 3 shows the construction process from word dependency to adjacency matrix. Specifically, we use Stanza to obtain the syntactic dependency tree of the sentence, and based on the syntactic dependency tree, we can construct the adjacency matrix *A* of the sentence. The adjacency matrix *A* is defined as shown in Eq. (11).

(11) \begin{eqnarray*}{A}_{i,j}= \left\{ \begin{array}{@{}l@{}} \displaystyle 1,\text{if}{c}_{i},{c}_{j}\text{has dependency}\\ \displaystyle 0,\text{otherwise} \end{array} \right. \end{eqnarray*}

where we set the main diagonal of the adjacency matrix *A* to 1 in order to preserve the information of its own nodes, and we construct the undirected graph, *i.e., A*_*i*,*j*_ = *A*_*j*,*i*_ , since we believe that the directed graph will lose some of the dependency information.

#### Graph convolutional neural network

We feed the adjacency matrix *A* constructed above into the graph convolutional network, which in this article is stacked in multiple layers, and then update the node representation in the next layer based on the hidden representation of the node’s neighborhood, with the graph convolutional formula shown in Eq. (12).

(12) \begin{eqnarray*}{h}_{i}^{l}=relu \frac{{A}_{i}{h}_{i}^{l-1}{W}^{l}}{D+1} +{B}^{l}\end{eqnarray*}

(13) \begin{eqnarray*}{D}_{i,j}= \left\{ \begin{array}{@{}l@{}} \displaystyle 1,{A}_{i,j}\not = 0\\ \displaystyle 0,{A}_{i,j}=0 \end{array} \right. \end{eqnarray*}

where *relu* is the nonlinear activation function, *W*^*l*^ is the linear transformation weight, *B*^*l*^ is the linear transformation bias term, $D={\mathop{\sum }\nolimits }_{j=1}^{n}{D}_{i,j}$ is the degree of *A*_*i*_ , and ${h}_{i}^{l-1}$ is the hidden representation of the GCN transformed from the previous layer.

#### Entity-specific masking mechanism

Above we capture the regional features of neighboring nodes by stacking multiple layers of GCNs, and to retrieve the semantic relationships of entities on their contextual words, we use an entity-specific masking mechanism, specifically, we mask the output non-entity vector of the last layer of GCNs with 0, while the vector representation of entities remains unchanged, as shown in Eq. (14).

(14) \begin{eqnarray*}Mas{k}_{i}^{l}=0 0\leq i\lt h,h+1\leq i\lt t,t+1\leq i\lt n\end{eqnarray*}

where *h* is the position index of the head entity, while *t* is the position index of the tail entity and *n* is the sentence length. Thus, we can obtain a representation of the entity-specific mask:

(15) \begin{eqnarray*}{h}_{mask}^{L}= \left\{ 0,\ldots ,{h}_{h}^{L},\ldots ,{h}_{t}^{L},\ldots ,0 \right\} .\end{eqnarray*}

Similar to Hong et al. (2021), we use the dot product attention mechanism to extract entity-specific features from contextual information and set the corresponding attention weights for each contextual word, and the attention weights are calculated under.

(16) \begin{eqnarray*}{\beta }_{i}=\sum _{j=1}^{n}{\mathbf{h}}_{i0}\top {h}_{j}^{L}\end{eqnarray*}

(17) \begin{eqnarray*}{\alpha }_{i}= \frac{\exp \nolimits \left( {\beta }_{i} \right) }{\sum _{j=1}^{n}\exp \nolimits \left( {\beta }_{j} \right) } .\end{eqnarray*}

The dot product can calculate the semantic correlation between entities and contextual words, which leads to the computation of the attention weight matrix, so that the final calculation of the input vector is shown in Eq. (18).

(18) \begin{eqnarray*}{R}_{gcn}=\sum _{i=1}^{n}{\alpha }_{i}{e}^{{c}_{i}}.\end{eqnarray*}

### Classification layer

Formally, we first fuse the local features extracted by PCNN and the global features focused on by the self-attention mechanism using the gating mechanism, and subsequently splice the fused features with the dependent features extracted by GCN, which in turn yields the final representation that is fed into the classification layer to produce the probability distribution p, as shown in Eqs. (19)–(20).

(19) \begin{eqnarray*}r= \left[ {R}_{gate};{R}_{gcn} \right] \end{eqnarray*}

(20) \begin{eqnarray*}p=fc \left( relu \left( {W}_{p}r+{b}_{p} \right) \right) \end{eqnarray*}

where ’;’ denotes the splicing of vectors, *fc* is the fully connected function, *W*_*p*_ and *b*_*p*_ are trainable parameters.

## Experiment

### Datasets and setup

This article evaluates the BERT-PAGG model on two public datasets: the SanWen dataset and the FinRE dataset, in which the SanWen dataset collects a corpus of 837 Chinese prose works, including 695 training sets, 58 validation sets and 84 test sets. The dataset includes 10 types of relations including “Unknown”. While the FinRE dataset collected 2,647 Sina financial news, and extracted 13,486 relationship instances as the training set, 3,727 relationship instances as the test set, and 1,489 relationship instances as the validation set, which contains 44 types of relationships.

In this article, a 768-dimensional word vector is generated using the BERT model with Adam as the optimizer and the learning rate set to 5e−5. To prevent overfitting, the dropout strategy is applied to the input and hidden layers in the training model and the dropout rate is set to 0.1. The hyperparameter settings of the BERT-PAGG model are shown in Table 3.

### Baselines

To evaluate the performance of the BERT-PAGG model for Chinese relationship extraction task, this article compares it with the following classical models.

• **Att-BLSTM**: Zhou et al. (2016) proposed a BLSTM model with word-level attention mechanism.
• **BLSTM**: A bidirectional LSTM model for relation extraction proposed by Zhang & Wang (2015).
• **PCNN**: A piecewise convolutional neural network model with multi-instance learning proposed by Le et al. (2018).
• **PCNN+Att**: An improved PCNN model with selective attention mechanism proposed by Lee, gyu Seo & Choi (2019).
• **Lattice LSTM**: A model for Chinese named entity recognition proposed by Zhang & Yang (2018), which explicitly utilizes word and word order information.
• **MG Lattice**: A model for Chinese relation extraction proposed by Li et al. (2019), which incorporates word-level information into character sequences and introduces external knowledge.
• **MGRSA**: Zhong (2021) proposed a Chinese entity relationship extraction model based on a multi-level threshold recursion mechanism and self-attention mechanism.

### Main results

As shown in Table 4, we report the performance of the BERT-PAGG model and the baseline model on two datasets, where the BERT-PAGG model is marked with “#” and “$” on both sides of the head and tail entities in the sample, respectively.

**Table 3:** Hyper parameters of BERT-PAGG model.

Parameter	Value
batch_size	64
epoch	10
lr	5e−5
optimizer	Adam
dropout	0.1
GCN-Layers	3

**Table 4:** Overall performance of different models on relationship extraction tasks.

Model	SanWen	FinRE
	Macro-F1	Macro-F1
Att-BLSTM	59.48	41.48
PCNN+Att	60.55	46.13
BLSTM	61.04	42.87
PCNN	61.20	45.51
Lattice LSTM	63.88	47.41
MG Lattice	65.61	49.26
MGRSA	67.12	52.61
ExSoftwords	70.90	—
BERT-PAGG	**73.83**	**53.01**

**Notes.**

The best results are in bold.

As can be seen from the above table, our proposed BERT-PAGG shows effectiveness and superiority in the relational extraction task compared to others, achieving a state-of-the-art Macro-F1 score. First, piecewise convolutional neural networks and self-attention mechanism can capture local features and global features well, while the addition of graph neural networks allows inter-word dependency features to be used effectively. Thus, we can observe that BERT-PAGG achieves the highest Macro-F1 value compared to the model using only sequence information.

### Ablation study

To investigate the effect of different components, we performed ablation experiments, and the results are shown in Table 5, where rows 1-4 are removing the specified components separately, in row 5, we use the lightweight Bert model, *i.e.,* Albert, to replace Bert to verify the effectiveness of BERT. From the Table 5, we can draw the following conclusions.

**Table 5:** The ablation study results.

Variants	SanWen	FinRE
	Macro-F1(%)	Macro-F1(%)
*M*_0_ : w/o Self-Attention	72.68	48.80
*M*_1_ : w/o PCNN	72.45	48.45
*M*_2_ : w/o Gate	70.76	48.27
*M*_3_ : w/o GCN	73.26	52.24
*M*_4_ : Only replace Bert with Albert	63.89	39.40
*M*_5_ : BERT-PAGG (full model)	**73.83**	**53.01**

**Notes.**

The best results are in bold.

1. The attention mechanism and the PCNN module can improve the feature extraction, thus improving the performance of the Chinese relationship extraction model.
2. The introduction of gating mechanism can better integrate the features extracted by the attention mechanism and PCNN module, and the gating mechanism can filter out the key features more wisely. Removing the gating mechanism reduces the BERT-PAGG model by at least 3% in the Macro-F1 metric, which indicates that the representation of text by PCNN and self-attention can be better obtained by the gating mechanism.
3. Without using inter-word dependency information, *i.e.,* removing the GCN module, the Macro-F1 of *M*_3_ is lower than that of *M*_5_ , this shows that the GCN module can effectively extract the dependency information between words and apply it to the relation extraction task. However, it is worth noting that the drop in Macro-F1 score is not significant after removing the GCN module, which may be due to the poor accuracy of the syntactic analyzer in the Chinese context, which in turn leads to errors in the construction of the adjacency matrix.
4. To verify the effectiveness of BERT for the Chinese relationship extraction task, *M*_4_ uses Albert with fewer parameters to replace BERT. Compared with *M*_5_, *M*_4_has a significant decline in Macro-F1, which indicates that BERT pre-training model can obtain more information from text and provide better text representation.

## Conclusion

In the experiments of relationship extraction, the PAGG module is designed in this article, which enables the model to incorporate entity location information and local features as well as dependency features, and pay more attention to key information, so that the BERT-PAGG relation extraction model proposed in this article achieves performance improvement compared with traditional relationship extraction models, such as LSTM, MG Lattice, PCNN, MGRSA, Att-BLSTM, etc. Meanwhile, to prove the effectiveness of the PAGG module designed in this article, the ablation experimental results show that the introduction of the PAGG module enhances the performance of relation extraction.

## Supplemental Information

10.7717/peerj-cs.1470/supp-1 Supplemental Information 1 Dataset

## Additional Information and Declarations

### Data Availability

The following information was supplied regarding data availability: The source code and data can be accessed at Zenodo: lishuaics, & Wenxuan Zhou. (2023). L-shuai/BERTPAG_GCN_final: v1.0.0 (dev). Zenodo. https://doi.org/10.5281/zenodo.7962611.
